# Differences in routine laboratory parameters related to cachexia between patients with hematological diseases and patients with solid tumors or heart failure – is there only one cachexia?

**DOI:** 10.1186/1475-2891-12-6

**Published:** 2013-01-07

**Authors:** Tomislav Letilovic, Sonja Perkov, Zlata Flegar Mestric, Radovan Vrhovac

**Affiliations:** 1 Department of Internal Medicine, Division of Cardiology, University Hospital Merkur, University of Zagreb School of Medicine, Zajceva 19, Zagreb, Croatia; 2 Department of Clinical Chemistry and Laboratory Medicine, University Hospital Merkur, Zajceva 19, Zagreb, Croatia; 3 Department of Internal Medicine, Division of Hematology, University Hospital Centre Zagreb, University of Zagreb School of Medicine, Kispaticeva 14, Zagreb, Croatia

**Keywords:** Cachexia, C-reactive protein, Albumin, Hemoglobin

## Abstract

**Background:**

Cachexia is a state of involuntary weight loss common to many chronic diseases. Experimental data, showing that cachexia is related to the enhancement of acute phase response reaction, led to the new definition of cachexia that included, aside from the principal criterion of weight loss, other “minor criteria”, Amongst them are levels of C-reactive protein (CRP), albumin and hemoglobin. However, there is paucity of data regarding possible differences of these laboratory parameters in patients with various diseases known to be related to cachexia.

**Methods:**

CRP, albumin and hemoglobin were evaluated in 119 patients, divided into two disease groups, hematological (ones with diagnosis of non-Hodgkin lymphoma or Hodgkin disease) and non-hematological (solid tumor patients and patients with chronic heart failure). Patients were further subdivided into two nutritional groups, cachectic and non-cachectic ones according to the principal criterion for cacxehia i.e. loss of body weight.

**Results:**

We found that cachectic patients had higher levels of CRP, and lower levels of both hemoglobin and albumin compared to non-cachectic patients, regardless of the disease group they fitted. On the other hand, the group of hematological patients had lower levels of CRP primarily due to the differences found in the non-cachectic group. Higher levels of albumin were also found in the hematological group regardless of the nutritional group they fitted. Limitations of cut-off values, proposed by definition, were found, mostly regarding their relatively low sensitivity and low negative predictive value.

**Conclusions:**

As expected, differences in values of routine laboratory parameters used in definition of cachexia were found between cachectic and non-cachectic patients. Their values differed between hematological and non-hematological patients both in cachectic and non-cachectic group. Cut-off levels currently used in definition of cachexia have limitations and should be further evaluated.

## Background

Cachexia is a state of involuntary weight loss common to many patients with chronic diseases such as cancer, acquired immunodeficiency syndrome, congestive heart failure, chronic infections, rheumatoid arthritis and kidney failure [1,2]. It was described centuries ago [3,4] and it is estimated that 5 million people in United States of America develop cachexia annually [5], with 2 million deaths relating to it [6] together with some other adverse prognostic features [7,8].

The mechanism of development of cachexia in all these conditions is believed to be similar if not the same [9]. Cachexia is believed to be a result of activation of acute phase response through upregulation of proinflammatory cytokines [10-12]. This upregulation is a consequence of various aforementioned diseases [13] and additionally, in cancer patients, cachexia is believed to be also induced by tumor products called proteolysis-inducing and lipid-mobilizing factors [14]. Those changes lead to alterations in metabolism of protein, fat and carbohydrates responsible for reduction in body weight as well as changes in body composition [15,16]. Upregulation of proinflammatory cytokines leads to elevated levels of C- reactive protein (CRP) and albumin [17,18] and chronic inflammation is also related to the development of anemia frequently referred as anemia of the chronic disease [19,20].

Relation between chronic inflammation, as a trigger for development of cachexia, and the subsequent changes in certain clinical and laboratory features lead key opinion leaders to incorporate, beside principal criterion for the diagnosis of cachexia which is at least 5% loss of edema – free body weight during the previous 12 months or less, other “minor criteria” into the new definition of cachexia. Amongst those “minor criteria” are elevated levels of CRP (≥ 5 mg/l) and decreased levels of hemoglobin (≤ 12 g/dl) and albumin (≤ 3.2 g/dl) [21] which were in the focus of our study.

Although it is well known that cachexia occurs in different diseases, comparative clinical or laboratory studies are lacking. Aim of the present study was to investigate whether there are differences between aforementioned routine laboratory parameters between patients with hematological diseases and other patients with diseases known to be related to cachexia (i.e. other solid tumors, chronic heart failure). First, we examined those parameters in all patients included in this study, thereafter investigating same parameters in cachectic and non-cachectic patients. Finally, we examined the proposed cut-off values of CRP, hemoglobin and albumin for diagnosis of cachexia, in order to establish their clinical usefulness.

## Methods

### Patients

The study was conducted at the Department of Internal Medicine of University Hospital Merkur in Zagreb. Patients' data were collected from May 2011 until February 2012 on consecutive patients that were eligible for entry. A diagnosis of either solid hematological tumors (non-Hodgkin lymphoma, Hodgkin disease), other solid tumors of any site or chronic heart failure of any etiology was mandatory. Diagnosis of malignant disease had to be proven with adequate histopathological sample. Diagnosis of heart failure was made according to the guidelines criteria [22].

Exclusion criteria were age of less than 18 years, starvation, malabsorption, diarrhea, active thyroid disease, depression or other severe psychiatric disease, chronic obstructive pulmonary disease, renal insufficiency of grade ≥ 3, myocardial infarction in less than last 12 weeks, liver insufficiency, neuromuscular diseases, alcohol or drug abuse and previous use of cardiotoxic chemotherapy in doses proven to be cardiotoxic [23].

The study received previous local ethical board approval and was conducted according to the Declaration of Helsinki and its subsequent amendments. All patients gave written informed consent before participation in the study.

### Evaluation of patients

Complete history was recorded, full physical examinations were performed and blood samples were collected at baseline. Baseline blood analyses included complete blood count and complete biochemistry (including values of hemoglobin, CRP and albumin). All measurements were done according to the standard protocol of institutional laboratory.

Patients were considered to be cachectic if they fulfilled main criteria for the diagnosis according to the consensus document. This criterion is loss of ≥ 5% of body weight in last 12 months or less (no less than 6 months). Other criteria (decreased muscle strength, fatigue, anorexia, low fat free index, abnormal values of CRP, hemoglobin or albumin), of which 3 of 5 need to be fulfilled for diagnosis, were also sought but we did not include them in the stratification of our patients [21].

Patients were further divided into two disease groups, hematological (ones with diagnosis of non-Hodgkin lymphoma or Hodgkin disease) and non-hematological (solid tumor patients and patients with chronic heart failure).

Measurements of CRP and albumin were performed on the Beckman Coulter Olympus AU 680 analyzer (Olympus Mishima Co., Ltd., Shizuoka, Japan) using Beckman Coulter System reagents and standardized laboratory methods on fresh sera on the day of blood collection. Serum was collected after centrifugation for 15 minutes at 3000 rpm. CRP concentrations were determined by automated high-sensitivity latex-enhanced immuno-turbidimetric assay. The calibrator CRP values were traceable to CRM 470. The limit of detection was 0.1 mg/L. Quantitative determination of albumin in serum was performed using Beckman Coulter bromocresol green photometric color test. Measurements of hemoglobin was performed on the Sysmex XE-2100 (Sysmex, Kobe, Japan), fully automated discrete hematology analyzer designed to generate complete blood counts. Hemoglobin determination was achieved using the sodium laurel sulfate (SLS)-hemoglobin method. Participation of Institute of Clinical Chemistry and Laboratory Medicine University Hospital Merkur accredited according to ISO 15189 (1,2) in the International External Quality Assessment Schemes for general and special medical biochemistry organized by World Health Organization for Laboratory Hematology (IEQAS - H) and by LABQUALITY, Finland for biochemical tests, confirms clinical reliability of obtained laboratory test results [24].

### Statistics

The results were expressed as the mean +/− standard deviation or as a proportion of the total number. Analyses of specificity, sensitivity, positive predictive value and negative predictive value were done using appropriate formulas. Differences in proportions (categorical variables) were compared using chi square test. Mann Whitney test was used to test the equality of continuous variables. A p-value < 0.05 was considered statistically significant. All statistics were performed with the StatView™ statistical program, version 5.0.1. (SAS Institute, Cary, NC, USA).

## Results

### Patients

One hundred nineteen (76 males; median age 62 years) consecutive patients were enrolled. Fifty five patients were from the hematological group (45 had diagnosis of non-Hodgkin lymphoma and 10 had diagnosis of Hodgkin disease). In the non-hematological group 43 patients were diagnosed with solid tumor of various sites (19 hepatocellular cancer, 8 colorectal cancer, 8 pancreatic cancer, 3 billiary duct cancer, 2 gastric cancer, 3 cancer of unknown primary site) and 21 had chronic heart failure (15 had ischemic cardiomyopathy and 6 had non-ischemic cardiomyopathy). In the group of heart failure patients 2 (10%) were in NYHA class I, 8 (38%) patients were in stage II, 9 (42%) were in stage III, and the remaining 2 (10%) in stage IV.

Hematological patients were significantly younger than non hematological patients. We found no statistically significant differences in prevalence of cachexia, lost body weight in cachectic patients or period of loss of body weight in cachectic patients between prespecified disease groups. Patient clinical characteristics are shown in Table 1.

**Table 1:** Clinical characteristics of patients

**Characteristic**	**Hematological**	**Non-hematological**	**p-value**
Number	55	64	
Age years (mean; SD)	56.85 (+/−14.58)	67.75 (+/−13.03)	p < 0.0001*
Men	37 (67%)	39 (61%)	NS
Cachectic (n/%)	15/27	27/35	NS
Lost body weight in kg in cachectic patients (mean; SD)	10.15 (+/−6.39)	11.51 (+/−7.25)	NS
Period of body weight loss in cachectic patients in months (mean; SD)	11.69 (+/−10.69)	14.53 (+/−17.93)	NS

Abbreviations: n = number, SD = standard deviation, kg = kilograms.

### Differences in values of CRP, hemoglobin and albumin between groups

Mean value of CRP in the whole group was 22.78 mg/l (+/−37.5). Hematological patients had mean value of CRP 15.98 mg/l (+/−36.1) and non-hematological patients had a mean value of CRP 28.71 mg/l (+/−39.1). Observed difference was found to be statistically significant (p = 0.048). When we subdivided patients according to their nutritional status, cachectic patients as a whole group had a mean CRP value of 34.18 mg/l (+/−51.9) and patients without cachexia had a mean value of CRP 17.57 mg/l (+/−27.5) which was also significantly different (p = 0.021). On the other hand, we could not find significant differences in CRP levels in hematological (32.90 mg/l (+/−64.5) vs. 9.75 mg/l (+/−12.9); p = 0.1) or in non-hematological (35.26 mg/l (+/−43.0) vs. 25.55 mg/l (+/−35.6); p=0.27) group when we divided them according to their cachectic status. In fact the observed initial difference was related to the difference between hematological and non-hematological patients in the non-cachectic group (9.75 mg/l (+/−12.9) vs. 25.55 mg/l (+/−35.6); p = 0.01). CRP levels according to the predefined groups are shown in Table 2.

**Table 2:** Values of CRP levels according to predefined groups

	**All**	**Cachectic**	**Non-cachectic**	**p-value (C vs. NC)**
All	22.78 (+/−37.5)	34.18 (+/−51.9)	17.57 (+/−27.5)	p = 0.021*
Hematological	15.98 (+/−36.1)	32.90 (+/−64.5)	9.75 (+/−12.9)	p = 0.1
Non-hematological	28.71 (+/−39.1)	35.26 (+/−43.0)	25.55 (+/−35.6)	p = 0.27
p-value (H vs. NH)	p = 0.048*	p = 0.43	p = 0.01*	

Abbreviations H = hematological, NH = non-hematological, C = cachectic, NC = non-cachectic. Values of CRP levels are in mg/l. *statistically significant.

(mean +/− standard deviation).

Mean value of hemoglobin in all examined patients was 12.53 g/dl (+/−2.1). Cachectic patients had lower levels of hemoglobin (11.73 g/dl (+/−2.0) vs. 12.90 g/dl (7.9-16.2); p = 0.039) than non-cachectic patients. There was no difference in values of hemoglobin between hematological and non-hematological patients (12.70 g/dl (+/−2.3) vs. 12.38 g/dl (+/−1.9); p = 0.33). All other analyzes done in the same way as for CRP values showed only statistically significant difference in hematological group depending on their nutritional status (11.20 g/dl (+/−2.2) vs. 13.27 g/dl (+/−2.0); p = 0.003). Hemoglobin levels according to the groups are shown in Table 3.

**Table 3:** Values of hemoglobin levels according to predefined groups

	**All**	**Cachectic**	**Non-cachectic**	**p-value (C vs. NC)**
All	12.53 (+/−2.1)	11.73 (+/−2.0)	12.90 (+/−2.0)	p = 0.039*
Hematological	12.70 (+/−2.3)	11.20 (+/−2.2)	13.27 (+/−2.0)	p = 0.003*
Non-hematological	12.38 (+/−1.9)	12.09 (+/−1.8)	12.53 (+/−2.0)	p = 0.27
p-value (H vs. NH)	p = 0.33	p = 0.16	p = 0.07	

Abbreviations H = hematological, NH = non-hematological, C = cachectic, NC = non-cachectic. Values of hemoglobin levels are in g/dl. *statistically significant.

(mean +/− standard deviation).

Mean level of albumin for all patients was 38.3 g/l (+/−6.5). Cachectic patients had lower levels of albumin (34.95 g/l (+/− 6.5) vs. 39.85 g/l (+/−5.8); p = 0.0002) both as a group and when divided into hematological (38.05 g/l (+/−6.1) vs. 42.04 g/l (+/−5.2); p = 0.019) and non-hematological ones (32.83 g/l (+/−6.1) vs. 37.85 g/l (+/−5.7); p = 0.004). On the other hand, hematological patients had higher level of albumin than non-hematological patients (40.95 g/l (+/−6.1) vs. 36.01 (+/−6.2); p = 0.0001) regardless of their nutritional status. Values of albumin levels are shown in Table 4.

**Table 4:** Values of albumin levels according to predefined groups

	**All**	**Cachectic**	**Non-cachectic**	**p-value (C vs. NC)**
All	38.31 (+/−6.5)	34.95 (+/−6.5)	39.85 (+/−5.8)	p = 0.0002*
Hematological	40.95 (+/−6.1)	38.05 (+/−6.1)	42.04 (+/−5.2)	p = 0.019*
Non-hematological	36.01 (+/−6.2)	32.83 (+/−6.1)	37.85 (+/−5.7)	p = 0.004*
p-value (H vs. NH)	p = 0.0001*	p = 0.02*	p = 0.0001*	

Abbreviations H = hematological, NH = non-hematological, C = cachectic, NC = non-cachectic. Values of albumin levels are in g/l. *statistically significant.

(mean +/− standard deviation).

### Evaluation of proposed cut-offs

Finally, we analyzed values of proposed cut-offs for CRP (≥ 5 mg/l), hemoglobin (≤ 12.0 g/dl) and albumin (≤ 32.0 g/l) both separately and together in the whole group of patients and upon dividing them into hematological and non-hematological groups. Sensitivities for the whole group were found to be 76% for CRP, 59% for hemoglobin, 35% for albumin and 86% for either of them being positive. Specificity was 39% for CRP, 68% for hemoglobin, 85% for albumin and 29% for either of them being positive. Negative predictive values were 78% for CRP, 79% for hemoglobin, 74% for albumin and 83% for all of them being negative. On the other hand, positive predictive value for CRP cut-off was 36%, for hemoglobin 46%, for albumin 52% and for either of them being positive 35%.

**Figure 1:**
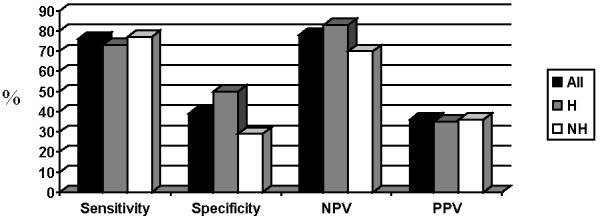
**Value of proposed cut-off for CRP (%).** Abbreviations: NPV = negative predictive value, PPV = positive predictive value, H = hematological, NH = non-hematological.

**Figure 2:**
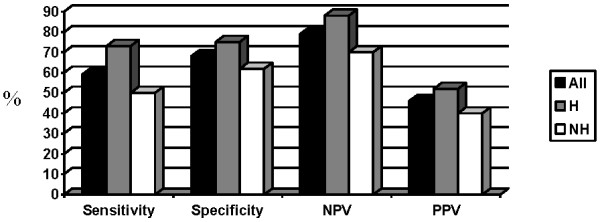
**Value of proposed cut-off for hemoglobin (%).** Abbreviations: NPV = negative predictive value, PPV = positive predictive value, H = hematological, NH = non-hematological.

**Figure 3:**
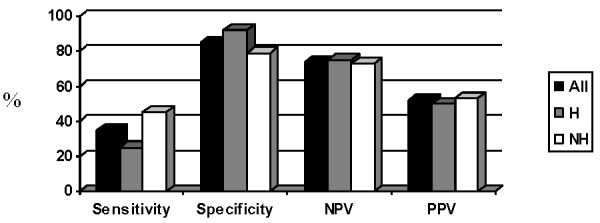
**Value of proposed cut-off for albumin (%).** Abbreviations: NPV = negative predictive value, PPV = positive predictive value, H = hematological, NH = non-hematological.

In hematological group, sensitivity of CRP was 73%, specificity 50%, negative predictive value was 83% and positive predictive value 35%. For hemoglobin, values were 73%, 75%, 88% and 52% and for albumin they were 25%, 92%, 75% and 50%, respectively. For all three values being either above or below proposed cut-offs values were 87%, 37%, 88%, 65%. Similar data were observed in non-hematological group and were for CRP for sensitivity 77%, 29% for specificity, 70% for negative predictive value and 36% for positive predictive value. For hemoglobin values were 50%, 62%, 70% and 40% and for albumin they were 45%, 79%, 73% and 53%. For all three cut-offs we got 92% sensitivity, 21% specificity, 75% negative predictive value and 45% of positive predictive value. Data for CRP, hemoglobin and albumin are shown in Figures 1, 2, 3.

Proposed cut-off for CRP did not significantly differentiate cachectic and non-cachectic patients neither in a whole group (p = 0.12) nor in hematological (p = 0.12) or non-hematological (p = 0.61) patients separately. Hemoglobin cut-off was able to discriminate cachectic and non-cachectic patients in the whole group (p = 0.043) and in hematological patients (p = 0.01) but not in non-hematological group of patients (p = 0.36). On the other hand, albumin cut-off discriminated well between cachectic and non-cachectic patients both in all patients (p = 0.011) and in non-hematological patients (p = 0.04), but not in hematological patients (p = 0.18). All three values being above or below the proposed cut-offs could not discriminate between cachectic and non-cachectic patients observed either together (p = 0.06) or separately as hematological (p = 0.08) or non-hematological (p = 0.44).

## Discussion

Before discussing further our results we believe that three possible questions, that may arise reading this paper, need to be clarified. Firstly, the rational to divide our patients into hematological and non-hematological group. Would it not have been more appropriate to classify them into those with tumor disease (hematological and solid tumors) and those with chronic benign state (chronic heart failure)? Would it not have been more appropriate to compare patients in the three separate groups i.e. hematological, solid tumors and heart failure group? There are several explanations for our decision. Our institution is a referral center for hematological diseases and most of the patients admitted to our institution indeed suffer from hematological diseases. Fewer patients are admitted for solid tumors, heart failure and other diseases related to cachexia. By dividing our patients in hematological and non-hematological ones we have achieved optimal balance of consecutive patients in respective groups. However, the answer is not just of technical or statistical matter. If one would be really interested, and it was indeed our intention, to investigate differences between hematological and patients with other diseases related to cachexia how is one supposed to divide patients? Dividing patients into three groups, apart from creating numerically unbalanced groups would not answer this question. In fact, in order to get the correct answer to this question a comparison between hematological patients and all other groups of patients with diseases that can cause cachexia would be necessary. Unfortunately, such a study could not have been conducted in our institution although we eagerly await a study by others that would address this question.

Secondly, we decided to divide patients into cachectic and non-cachectic just according to the main criterion that is loss of at least 5% of body weight during the previous 12 months or less, not further looking at other criteria proposed by new definition that we refer to [21]. We think that such, at first glance, arbitrary division is founded on the majority of the existing experimental data that defined cachexia just as s state with a certain amount of weight loss during variable time frame. Although we do not question the new definition we still think it can be subjected to various tests. One of them could be determining relations between its major and minor criteria. Comparing results of minor criteria upon establishing groups of patients after using major criteria is just that. In accordance with our work also goes the new proposed definition for cancer cachexia which states that only the loss of weight of 5% for 6 months is a criterion enough to diagnose cancer cachexia [25].

And lastly we decided, as is obvious from our patient characteristics, to enroll patients that had weight loss for more than 12 months. We think that it is justifiable, although the definition that we refer to [21] states the weight loss should be in 12 months or less. But the authors also state that “*… .time frame may be disease specific and is likely to be shorter in cancer (3–6 months) and longer in chronic heart failure and kidney disease (12 months)… . .*”. Although this sentence may imply the upper limit of 12 months we believe that it also may imply periods longer than 12 months. Some of the published data refers to periods exceeding 12 months, as was the case in one of the pivotal studies from Anker et al. with duration of weight loss at inclusion up to 13 months. After inclusion patients were followed for a mean of 686 days which is almost two years [26]. In that study, patients that exceeded 12 months of body weight loss after inclusion were not excluded from follow up and we have followed the same reasoning in our investigation. It is especially important in patients with durable survival in spite of their disease (hematological patients with lymphomas, some cancer patients, heart failure patients) so periods of loss of body weight can be longer.

Given the scarcity of data regarding clinical and laboratory features comparing patients with various diseases that are related to cachexia we conducted our study to address some of these issues. We were specifically focused on differences between hematological and non-hematological patients. We found, as others did, that cachectic patients as a group, had significantly higher levels of CRP and lower levels of both albumin and hemoglobin. Secondly, we divided patients into hematological and non-hematological groups and found that hematological patients had significantly lower levels of CRP and higher levels of albumin. There was no difference in levels of hemoglobin. When we further subdivided patients into cachectic and non-cachectic groups we found that the differences observed for CRP were mainly due to differences found in non-cachectic group. Observed differences in albumin levels were present regardless of this subdivision. Hematological cachectic patients had lower levels of hemoglobin than non-cachectic ones. The same was not true for non-hematological patients. Observed differences could imply possible divergences in pathophysiology between the groups of patients as we divided them. Unfortunately, we were not able to conduct investigations regarding possible differences in for example expression of inflammatory cytokines that could explain observed differences. Because of that our question “is there only one cachexia?”, although provocative, remains purely speculative and awaits further trials with larger numbers of patients and more balanced groups exploring other features of cachexia, probably primarily those incorporated into proposed definition [21]. New proposed definition orientating just on cancer cachexia is in the line with our work i.e. it already recognizes the existence of “different” types of cachexia [25].

We then evaluated proposed cut-offs for CRP (≥ 5mg/L), hemoglobin (≤ 12 g/dl) and albumin (≤ 3.2 g/dl) and found that CRP had highest sensitivity, followed by hemoglobin and albumin. Inverse relationship was found for specificity. Cut-offs were found to have relatively good negative predictive value whereas positive predictive value was poor. Combining all three cut-offs just emphasized above mentioned relations. When we divided patients to the hematological and non-hematological groups, proposed cut-offs followed same trends as in the whole group. Proposed cut-off for CRP was not able to distinct cachectic from non-cachectic patients in the whole group nor was it able to distinct them when we further subdivided them into hematological and non-hematological groups. On the other hand, proposed cut-off for albumin was able to distinct patients according to their nutritional status regardless of further subdivisions. Hemoglobin cut-off showed its predictive power in both whole group as well as in hematological group but not in non-hematological group. Although such results would imply that CRP cut-off is least effective and albumin cut-off is the most effective one such conclusions would need larger studies powered enough even to give more precise values. Until then, our results serve as ones that arouse suspicion in proposed cut-offs.

## Conclusions

Patients suffering from diseases related to cachexia have elevated values of routine laboratory parameters related to enhanced inflammation. We found significant differences between these values for cachectic and non-cachectic groups of patients. We also revealed some significant differences between these values when dividing patients into hematological or non-hematological groups, although we are well aware of the relatively small number of patients in groups and of the variability of patients’ diagnoses especially in the group of patients with solid tumors. We are also aware of the difference in age between the hematological and non-hematological group that could have influenced our results. These restrictions stopped us from attributing observed differences to differences in pathophysiological mechanisms of exaggerated immune response in different diseases. On the other hand, limitations of our study certainly cannot allow anyone to conclude that those differences in fact actually do not exist. To our knowledge, this is the first study that tries to compare between groups of patients suffering from the different diseases that can cause cachexia. Further studies with larger numbers of patients and with smaller variability inside the groups are warranted for further clarification of possible differences between the cachexia presenting in various diseases.

## Abbreviations

CRP: C- reactive protein; H: Hematological; NH: Non-hematological; C: Cachectic; NC: Non-cachectic.

## Competing interests

The author’s declare that they have no competing interests.

## Authors’ contributions

TL gathered all the patients’ data and blood samples and participated in the design of the study and statistical analyses. SP and ZFM conducted laboratory analyses vital to the study. RV participated in the design of the study and statistical analyses. All authors read and approved the final manuscript.
